# Wound Infection

**Published:** 1895-03-09

**Authors:** 


### Wound Infection.

In the treatment of wounds we endeavour to close every route against the entrance of the microbe, and this in a word is what is meant by their aseptic management. But in dealing with a wound which has once become septic the case is different, for the microbe has already gained admission. Here, in spite of much that has been lately said to the contrary, there is reason still to believe that a great deal may be done by the local application of germicides, and by antiseptic irrigation at the seat of infection.

No doubt certain septic bacteria are very rapidly absorbed from fresh wounds, and five minutes after introduction they may be found in the internal viscera. If a mouse, for example, be inoculated at the tip of its tail with anthrax, amputation of the tail ten minutes after does not save the mouse.

But, in regard to ordinary wound infection in man, it is not a question of anthrax, and the impossibility of preventing this disease in certain animals by the immediate application of antiseptics is no proof that in man ordinary pyogenic bacteria may not be rendered harmless by such a process. Nay, the very fact that man does at times suffer from a purely localised form of anthrax shows that the law of rapid general infection does not hold good in him as it does in certain animals, even in regard to that disease.

It must be understood that the bacteria which generally cause wounds to inflame in animals are different from those which give rise to the same condition in man; in fact, each species of animal seems to have a bacterium peculiar to itself, but it has been found that a phlegmonous process in man may be transferred to certain of the lower animals, and produce in them a phlegmonous inflammation. The difference in virulence of the various bacteria is believed to depend upon their power of resistance to the germicidal action of the tissues of the body, and whilst some can develop perfectly well in the ordinary juices of the body, others can only thrive where they are able to cause the destruction of a given area. Every wound is more or less a *locus minoris resistentiæ*, and may thus prove a suitable soil for the growth of bacteria of low resisting power, which, in uninjured tissue, would be quite unable to develop. The diminished resistance of the tissues is the microbe's opportunity, and how far we can aid these tissues against the invader may be shown by reference to some recent investigations. Rabbits' ears, infected with streptococcus pus, were irrigated with 1:1000 mercuric solution or 4 per cent. carbolic, and it was found that two hours after infection the disinfection had full effect, and, when employed six hours after, the severity of the infection was greatly diminished. Again, experiments have been made with a 3 per cent. lysol or carbolic solution. In these two sets of animals were selected; in each case, as nearly alike as possible in every respect, and both were infected in the same way and the wound dressed with dry aseptic dressing. After a certain time the wound in one set of the animals was washed out with sterilised salt solution, and again covered with dry aseptic dressing. The wound in the other was washed with a 3 per cent. lysol or carbolic solution at 37 degrees Fahr. The disinfected wound was then loosely packed with wet carbolic gauze, &c., covered with a wet carbolic dressing.

Of the ten rabbits treated aseptically all but one died, in from eight to fourteen days, of extensive phlegmonous suppuration, while of the ten rabbits treated antiseptically all but one recovered. In two of those treated antiseptically the wounds healed without suppuration. In only one was there extensive suppuration. In the others there occurred moderate suppuration, but this was entirely limited to a narrow area immediately around the wound. Inoculations, made with pus from these two sets of animals, gave very interesting results. All the animals inoculated from the aseptically treated wounds perished in from twenty-four to forty-eight hours, while those infected from the antiseptically treated wounds all lived.

The importance of these experiments lies in their showing that the results of the mouse-tail experiments in regard to anthrax cannot be accepted as disproving the efficacy of disinfectants in regard to pyogenic organisms, for they demonstrate that it is possible, at any rate in rabbits, to thoroughly disinfect a wound by antiseptic irrigation and dressing, and that if suppuration is not always prevented thereby, its sphere is so limited that it assumes a purely local character.

It is interesting to note that a 3 per cent. carbolic solution in no way lessens the resisting power of the tissues against pyogenic cocci, nor does it predispose them to suppuration. Indeed, the very opposite appears to be the case. There is no doubt that the further we advance in our knowledge of wounds and their treatment, the greater must be our caution as to the direction our next step shall take.

Experiment is doubtless the basis of knowledge, but every experiment should tally with experience, which is but experiment on a larger sale. Truth must be the same in the ward and in the laboratory, and it is satisfactory to find that the trust which surgeons have learned to place in antiseptics is supported by these experiments, which tend to prove their efficacy against the microbic infection usually met with.

